# *phoP*, SPI1, SPI2 and *aroA* mutants of *Salmonella* Enteritidis induce a different immune response in chickens

**DOI:** 10.1186/s13567-015-0224-x

**Published:** 2015-09-17

**Authors:** Marta Elsheimer-Matulova, Karolina Varmuzova, Kamila Kyrova, Hana Havlickova, Frantisek Sisak, Masudur Rahman, Ivan Rychlik

**Affiliations:** Veterinary Research Institute, Hudcova 70, 621 00, Brno, Czech Republic

## Abstract

Poultry is the most frequent reservoir of non-typhoid *Salmonella enterica* for humans. Understanding the interactions between chickens and *S. enterica* is therefore important for vaccine design and subsequent decrease in the incidence of human salmonellosis. In this study we therefore characterized the interactions between chickens and *phoP*, *aroA*, SPI1 and SPI2 mutants of *S*. Enteritidis. First we tested the response of HD11 chicken macrophage-like cell line to *S*. Enteritidis infection monitoring the transcription of 36 genes related to immune response. All the mutants and the wild type strain induced inflammatory signaling in the HD11 cell line though the response to SPI1 mutant infection was different from the rest of the mutants. When newly hatched chickens were inoculated, the *phoP* as well as the SPI1 mutant did not induce an expression of any of the tested genes in the cecum. Despite this, such chickens were protected against challenge with wild-type *S*. Enteritidis. On the other hand, inoculation of chickens with the *aroA* or SPI2 mutant induced expression of 27 and 18 genes, respectively, including genes encoding immunoglobulins. Challenge of chickens inoculated with these two mutants resulted in repeated induction of 11 and 13 tested genes, respectively, including the genes encoding immunoglobulins. In conclusion, SPI1 and *phoP* mutants induced protective immunity without inducing an inflammatory response and antibody production. Inoculation of chickens with the SPI2 and *aroA* mutants also led to protective immunity but was associated with inflammation and antibody production. The differences in interaction between the mutants and chicken host can be used for a more detailed understanding of the chicken immune system.

**Electronic supplementary material:**

The online version of this article (doi:10.1186/s13567-015-0224-x) contains supplementary material, which is available to authorized users.

## Introduction

Non-typhoid *Salmonella enterica* serovars are among the most common causative agents of food-borne diseases worldwide [1]. Since poultry is the most frequent reservoir of salmonellosis for humans, vaccination of chickens is understood as an effective measure to decrease *S. enterica* incidence in humans. Currently, construction of attenuated vaccine strains of *S. enterica* is not an issue and many different mutants have been tested in mice, chickens and even humans [2-7]. However, the main dilemma is which mode of attenuation to choose out of the many possibilities [8]. More detailed information on host response to *S. enterica* infection or vaccination is therefore needed. Such information can be obtained either by generating chickens with knocked out genes involved in innate or acquired immune response or by preparing *S. enterica* mutants with clearly defined defects in pathogenesis and analysis of chicken immune response. Since the former possibility is still an issue in chickens, the latter approach represents a feasible alternative.

Mutants with clearly different defects in *Salmonella* pathogenesis include those with deletions in *aroA*, *phoP*, SPI1 or SPI2. Reduced virulence of *aroA* mutants can be explained by their inability to produce aromatic compounds as well as having a high sensitivity to serum [2,9]. *phoP* mutants belong to the most attenuated ones as they fail to survive inside phagocytic cells [10], perhaps due to their high sensitivity to acidification and host antimicrobial peptides [11]. However, *phoP* mutants also exhibit intracellular overgrowth in fibroblasts [12]. Recently, mutants defective in virulence genes specific to *S. enterica* such as those localized on the *Salmonella* pathogenicity island (SPI) 1 and SPI2 have been successfully tested [5,13]. SPI1 mutants are impaired in invading non-professional phagocytes while SPI2 mutants are unable to survive intracellularly for a prolonged time [14-17]. SPI1 mutants are also defective in induction of apoptosis in macrophages [18,19]. Interestingly, when we recently used SPI1 and SPI2 mutants of *S. enterica* serovar Enteritidis for vaccination of chickens, higher antibody levels were observed in chickens vaccinated with the SPI2 mutant than in chickens vaccinated with the SPI1 mutant [13]. Inactivation of different branches of *S. enterica* virulence may therefore lead to its different recognition by the chicken immune system and induction of a different type of specific immunity.

Comparison of chicken response to inoculation with different *S. enterica* mutants is further complicated by the fact that with increasing age, chickens become quite resistant to *S. enterica* infection [20]. Consequently, although there are numerically lower counts of *Salmonella* in the liver and spleen, and lower inflammatory responses are recorded in 6-week-old vaccinated chickens in comparison with non-vaccinated controls after challenge, such differences do not always reach statistical significance with the numbers of chickens commonly used under laboratory conditions. This was the reason why we recently initiated research activities using genomic and proteomic tools which led to the identification of tens of genes whose expressions change after *S*. Enteritidis infection of newly hatched chickens [21,22]. Some of them can be induced by *S*. Enteritidis infection even in 42-day-old chickens [21], although our subsequent study indicated that induction of these genes in 42-day-old chickens might not be as reliable as we initially expected [23]. In this study we therefore first characterized the response of chicken macrophage cell line HD11 to infection with wild-type *S.* Enteritidis and *aroA*, *phoP*, SPI1 and SPI2 mutants, as macrophages are considered to play a key role in the immune response to *Salmonella* infection. In the second part of this study we performed in vivo experiments and compared the type of immunity induced by oral inoculation of newly hatched chickens with wild-type *S.* Enteritidis and its mutants. We found out that the SPI1 or *phoP* mutants stimulated protective immunity without inducing inflammation and immunoglobulin production in vivo in the chicken cecum. *aroA* or SPI2 mutants also induced protective immunity, however, inoculation of chickens with these mutants resulted in moderate inflammation and antibody production.

## Materials and methods

### Bacterial strains and in vitro testing in HD11 cells

*S*. Enteritidis 147 spontaneously resistant to nalidixic acid with a proven virulence in chickens and mice [6,24] was used in this study. All isogenic mutants had been constructed earlier and are listed in Table 1. Chicken macrophage-like cell line HD11 was cultured at 37 °C under 5% CO_2_ atmosphere in RPMI-1640 (Sigma). Bacteria were grown statically in LB broth at 37 °C for 18 h. This culture was diluted 800× in LB broth and incubated for an additional 3 h at 37 °C to obtain bacteria in the late logarithmic growth-phase of a highly invasive phenotype. Prior to infection of HD11, the bacteria were pelleted by centrifugation (10 min at 6500 × *g*) and re-suspended in PBS to OD = 0.3. HD11 cells were infected with *S.* Enteritidis or its mutants at a multiplicity of infection equal to 1 for 1 h. Free bacteria were then washed away and gentamicin was added to fresh RPMI-1640 medium (100 μg/mL) to kill any remaining extracellular bacteria. One hour later, the medium was replaced with fresh medium containing 15 μg/mL gentamicin to prevent multiplication of extracellular bacteria that were eventually released during culture from dead cells. Two and 22 h later, i.e. 4 and 24 h after the infection of HD11 cells, the appropriate number of wells were either lysed with 1% Triton X-100 to release intracellular bacteria or treated with TRI Reagent for RNA purification (see below). Serial decimal dilutions were plated on LB agar plates to count released bacteria. The whole experiment was performed in duplicates on two independent occasions.

**Table 1:** ***Salmonella*** **Enteritidis strains used in the study**

**Strain**	**Resistance** ^**1**^	**Reference**
*S*. Enteritidis 147	Nal	[6]
*S.* Enteritidis 147 ΔSPI1	Nal	[6]
*S.* Enteritidis 147 ΔSPI2	Nal	[6]
*S.* Enteritidis 147 Δ*aroA*::Cm	Nal, Cm	[9]
*S*. Enteritidis 147 Δ*phoP*::Cm	Cm	[46]

^1^Nal, nalidixic acid; Cm, chloramphenicol.

### In vivo experimental design and sample collection

Male ISA Brown chickens (Hendrix Genetics, the Netherlands) were obtained from a local commercial hatchery on day of hatch. Chickens were reared in perforated plastic boxes with free access to water and feed. Each experimental or control group was kept in a separate room.

In the first experiment, 4 newly hatched chickens per group were orally inoculated with 0.1 mL of wild-type *S*. Enteritidis 147 and SPI1, SPI2, *aroA* or *phoP* mutants. Infectious dose was approx. 10^8^ CFU and infected chickens were euthanized 4 days post infection (dpi). The control group consisted of 4 non-infected chickens euthanized on day 5 of life. During necropsy, approx. 30 mg of the cecum was collected from each chicken, placed into RNALater (Qiagen) and kept at −70 °C prior to RNA isolation.

In the second experiment, 4 chickens were orally infected on day of hatch (day 1), on day 22 or day 43 of life with approx. 10^8^ CFU of *S*. Enteritidis 147. Infected chickens were euthanized 4 dpi. Four age-matched non-infected control chickens were also included. During necropsy, cecum samples were collected into RNALater and kept at −70 °C.

In the third experiment, 6 chickens per group were orally inoculated with wild-type *S*. Enteritidis 147 and SPI1, SPI2, *aroA* or *phoP* mutants on day 1 of life, orally challenged with 10^8^ CFU of the wild-type *S*. Enteritidis on day 22, and euthanized 4 days later. Six age-matched, non-infected control chickens and 6 non-inoculated but challenged chickens were also included in this experiment.

In the last experiment we verified results from the first and third experiment. Sixteen chickens per group were orally inoculated with wild-type *S*. Enteritidis 147 and SPI1, SPI2, *aroA* or *phoP* mutants on day 1 of life. Six chickens from each group were euthanized 4 days post inoculation, another six chickens from each group were euthanized prior to challenge on day 22 of life. The remaining chickens were challenged on day 22 of life and euthanized 4 days post challenge. Non-infected control chickens sacrificed on day 5 and 26 of life (4 chickens per each time point), and 4 non-inoculated chickens challenged on day 22 of life and sacrificed 4 days later were included as well. Since the same experimental set up was used in the experiments 1, 3 and 4, data from these are combined in all figures or tables as appropriate.

All animal treatment and handling was performed in accordance with the current Czech legislation (Animal protection and welfare Act No. 246/1992 Coll. of the Government of the Czech Republic) and has been approved by the Ethics Committee for Animal Welfare of the Ministry of Agriculture of the Czech Republic (permit number MZe 1226).

### Bacteriology

Approx. 0.5 g of liver tissue and cecal content was collected from chickens during necropsy performed after all experiments. The samples were homogenized in peptone water, tenfold serially diluted and plated on XLD agar plates (HiMedia) supplemented with nalidixic acid, or Brilliant Green Agar (Oxoid) supplemented with chloramphenicol in the case of the *phoP* mutant. Detection limit of direct plating was 500 CFU/g of sample. Samples negative after direct plating were subjected to enrichment in modified semi-solid Rappaport-Vassiliadis medium (Oxoid) for qualitative *S*. Enteritidis counts determination. Counts of *S*. Enteritidis found positive after direct plating were logarithmically transformed. Samples found positive only after enrichment were assigned a value of 1 and negative samples were assigned a value of 0.

### RNA purification, reverse transcription and quantitative RT-PCR

Cecal samples were recovered from RNALater storage, mixed with 1 mL TRI Reagent (MRC) and homogenized with MagNALyzer (Roche). Fifty μL of bromoanisole was added to the homogenate and after centrifugation for 15 min at 14 000 × *g*, the upper phase containing RNA was collected and purified with RNeasy Mini Kit (Qiagen). The concentration and purity of RNA was determined spectrophotometrically (Nanodrop, Thermo Scientific). One μg of RNA was immediately reverse transcribed into cDNA using M-MLV reverse transcriptase (Invitrogen) and oligo(dT) primers. Following the reverse transcription, the cDNA was diluted 10× with sterile water and stored at −20 °C prior to quantitative real-time PCR.

Mucosal immune response was characterized by real-time PCR based on the expression of 36 genes identified earlier [21,22]. Primers for the quantification of gene expression by real-time PCR are listed in Additional file 1. Real-time PCR was performed in 3 μL volumes in 384-well microplates using QuantiTect SYBR Green PCR Master Mix (Qiagen) and Nanodrop II Stage pipetting station (Innovadyne) for PCR mix dispensing. The amplification and signal detection were performed using a LightCycler II (Roche) with an initial denaturation at 95 °C for 15 min followed by 40 cycles of 95 °C for 20 s, 60 °C for 30 s and 72 °C for 30 s. Each sample was subjected to real-time PCR in a duplicate and the mean Ct value of duplicates was used for subsequent calculations. The Ct values of the genes of interest were normalized (ΔCt) to an average Ct value of three house-keeping genes, i.e. glyceraldehyde-3-phosphate dehydrogenase (GAPDH), TATA box binding protein (TBP) and ubiquitin (UB). The relative expression of each gene of interest was then calculated as 2^−ΔCt^.

### Statistical analyses

*Salmonella* counts in HD11 cells, chicken tissues and gene expression data from real-time PCR were analyzed with ANOVA test followed by *post hoc* Tukey’s multiple comparison test. *P* values ≤ 0.05 were considered as significant. Heat maps were constructed in R using gplots package with values standardized to row Z-scores. Experimental groups in heat maps were reordered according to column means.

## Results

### Infection of HD11 cells

**Figure 1:**
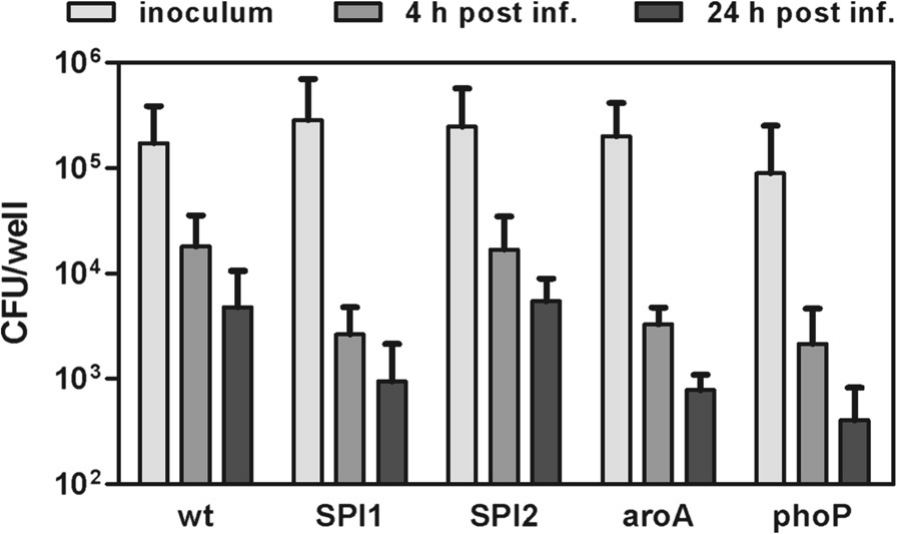
**Invasion and intracellular survival of the wild-type** ***S.*** **Enteritidis and its mutants in HD11 cells.** HD11 cells were inoculated with approx. 10^5^ CFU *S.* Enteritidis wild-type or the SPI1, SPI2, *aroA* or *phoP* mutant (MOI = 1). Four and 24 h post infection, intracellular bacterial counts were determined. Bars represent mean and SD.

First we tested whether the mutants and wild-type *S*. Enteritidis will differently interact with chicken macrophage-like cell line in vitro being aware that the interaction with HD11 cell line may not directly correlate with the interaction of the strain with chicken immune system in vivo. SPI1, *aroA* and *phoP* mutants were present at lower intracellular counts than the wild-type *S*. Enteritidis or the SPI2 mutant 4 h post infection (Figure 1) but none of the comparisons reached statistical significance. Twenty four hours post infection, intracellular counts of the wild-type strain and the mutants decreased 3 to 5 fold.

**Figure 2:**
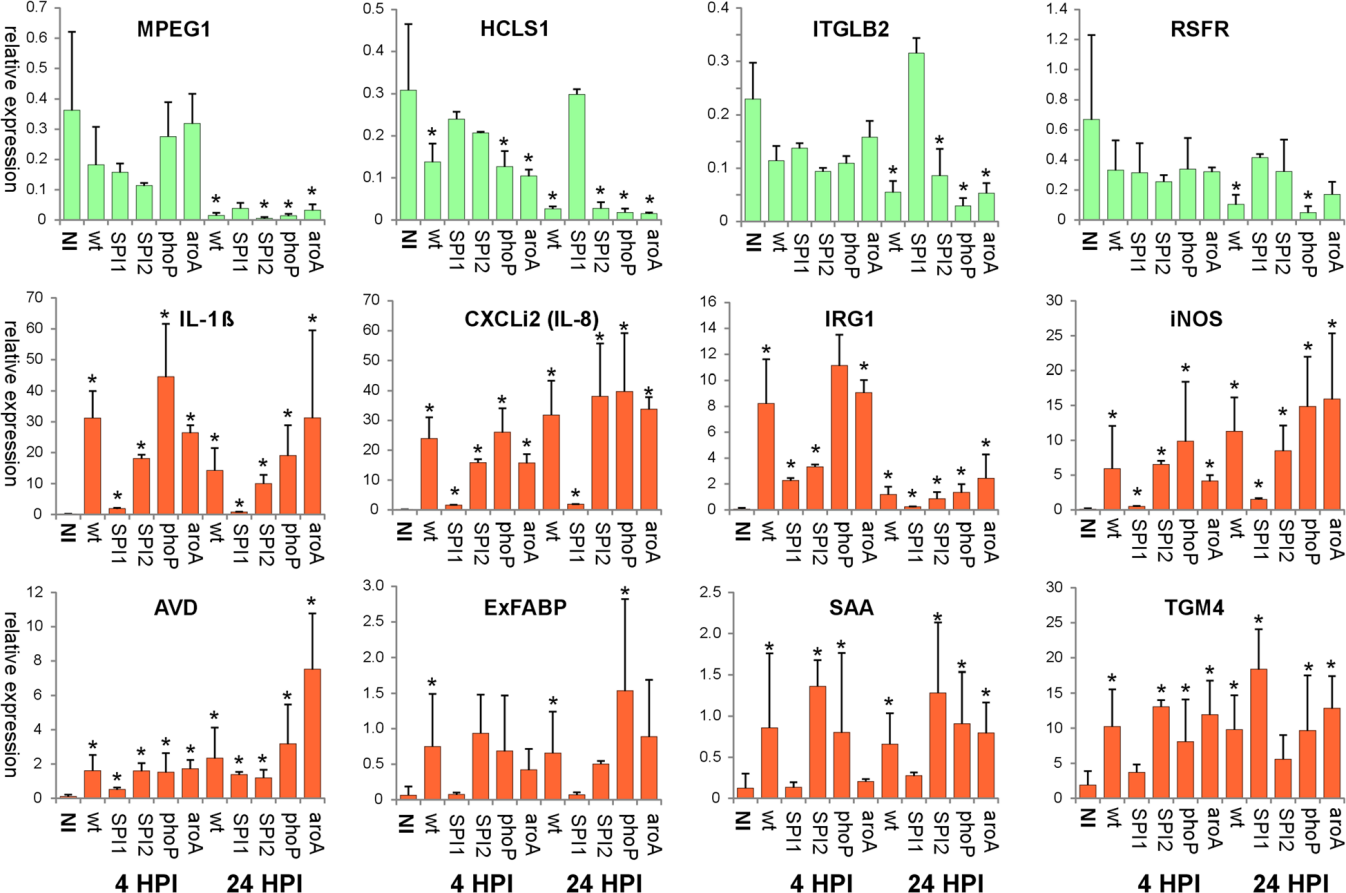
**Expression of genes in HD11 cells inoculated with wild-type** ***S.*** **Enteritidis and its mutants.** The SPI1 mutant stimulated a different response in HD11 cell line than the remaining strains or mutants. Panels in green, genes suppressed by *S*. Enteritidis infection 24 hours post infection (hpi). Panels in red, genes induced by *S*. Enteritidis infection. Bars represent expression normalized to the average expression of 3 house-keeping genes. * - statistically significant difference from the expression in the non-infected cells at *P* ≤ 0.05.

When gene expression was determined, 14 genes out of 36 tested were considered as not expressed in HD11 cells as their relative expression was lower than 0.05, i.e*.* their expression did not reach 5% of the expression of the house-keeping genes (data not shown). Genes such as TRAP6, AH221, STAT3, C3, ASL2, STAT1, EPSTI1, IFIT5, RSFR, MPEG1, ITGLB2 and HCLS1 were expressed in HD11 cells but though inducible in the chicken cecum following *S*. Enteritidis infection [22], these were not induced HD11 macrophages in response to *S*. Enteritidis infection. Instead, RSFR, MPEG1, ITGLB2 and HCLS1 were suppressed in HD11 cells 24 h after infection with wild type *S*. Enteritidis. These genes were usually suppressed also after infection with the SPI2, *phoP* and *aroA* mutants but not with the SPI1 mutant (Figure 2).

The last group of genes included IL-1β, CXCLi2 (IL-8), AVD, IRG1, iNOS, ExFABP, TGM4 and SAA whose expression in HD11 cells increased after the infection with *S*. Enteritidis (Figure 2). IL-1β, CXCLi2 (IL-8), AVD, IRG1 and iNOS were induced by all the strains and at both time points. Significant induction was less frequent for ExFABP, TGM4 and SAA due to their lower induction rate in comparison to IL-1β, CXCLi2 (IL-8), AVD, IRG1 and iNOS. However, the only strain which never significantly induced ExFABP, TGM4 and SAA in HD11 macrophage cell line was the SPI1 mutant (Figure 2).

### Chicken response to inoculation with SPI1, SPI2, *phoP* and *aroA* mutants of *S.* Enteritidis

**Figure 3:**
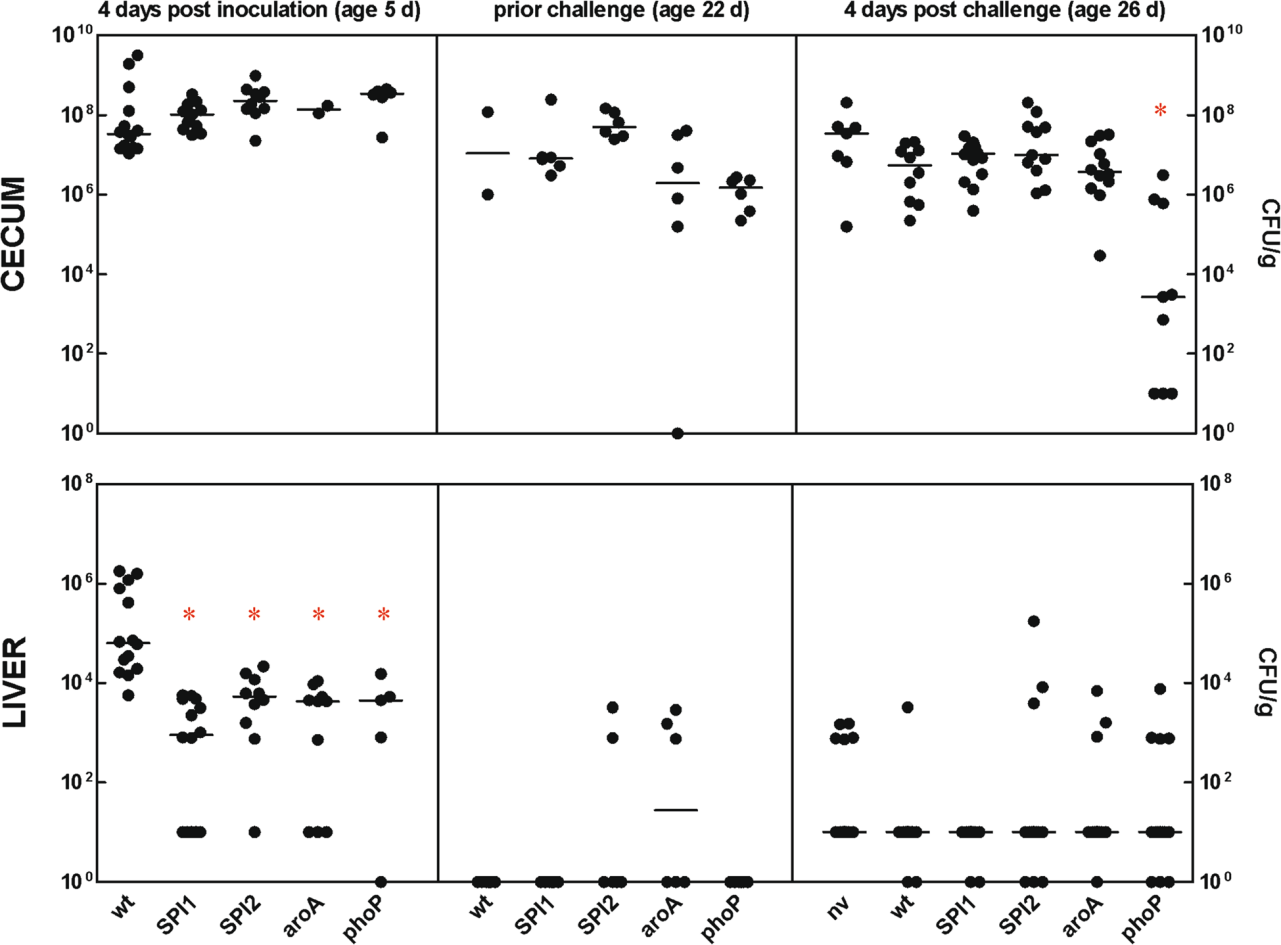
***Salmonella*** **counts in organs of chickens inoculated with wild-type** ***S.*** **Enteritidis and its mutants.** Chickens were inoculated on day of hatch with SPI1, SPI2, *phoP* and *aroA S*. Enteritidis mutants and challenged on day 22 of life. The data represent individual values and median CFU/g tissue. Asterisks indicate statistically significant differences from chickens inoculated with wild-type *S.* Enteritidis at *P* ≤ 0.05 (*). Non-vaccinated chickens infected on day 22 of life are designated as “nv”. Some values from the chicken cecum are missing due to an overgrowth of plates with non-*Salmonella* microbiota resistant to nalidixic acid.

As there were differences in the gene expression response of HD11 macrophages to the infection with different mutants, in the next experiment we tested whether chickens would also recognize and respond differently to inoculation with 4 different mutants and wild-type *S*. Enteritidis. Four days after the inoculation, SPI1, SPI2, *phoP* and *aroA* mutants colonized the cecum similarly as wild-type *S*. Enteritidis and *Salmonella* counts in the cecum in all inoculated groups were around 10^8^ CFU/g (Figure 3). However, systemic spread of all 4 mutants was limited as their counts in the liver were significantly lower than that of the wild-type strain (Figure 3).

**Figure 4:**
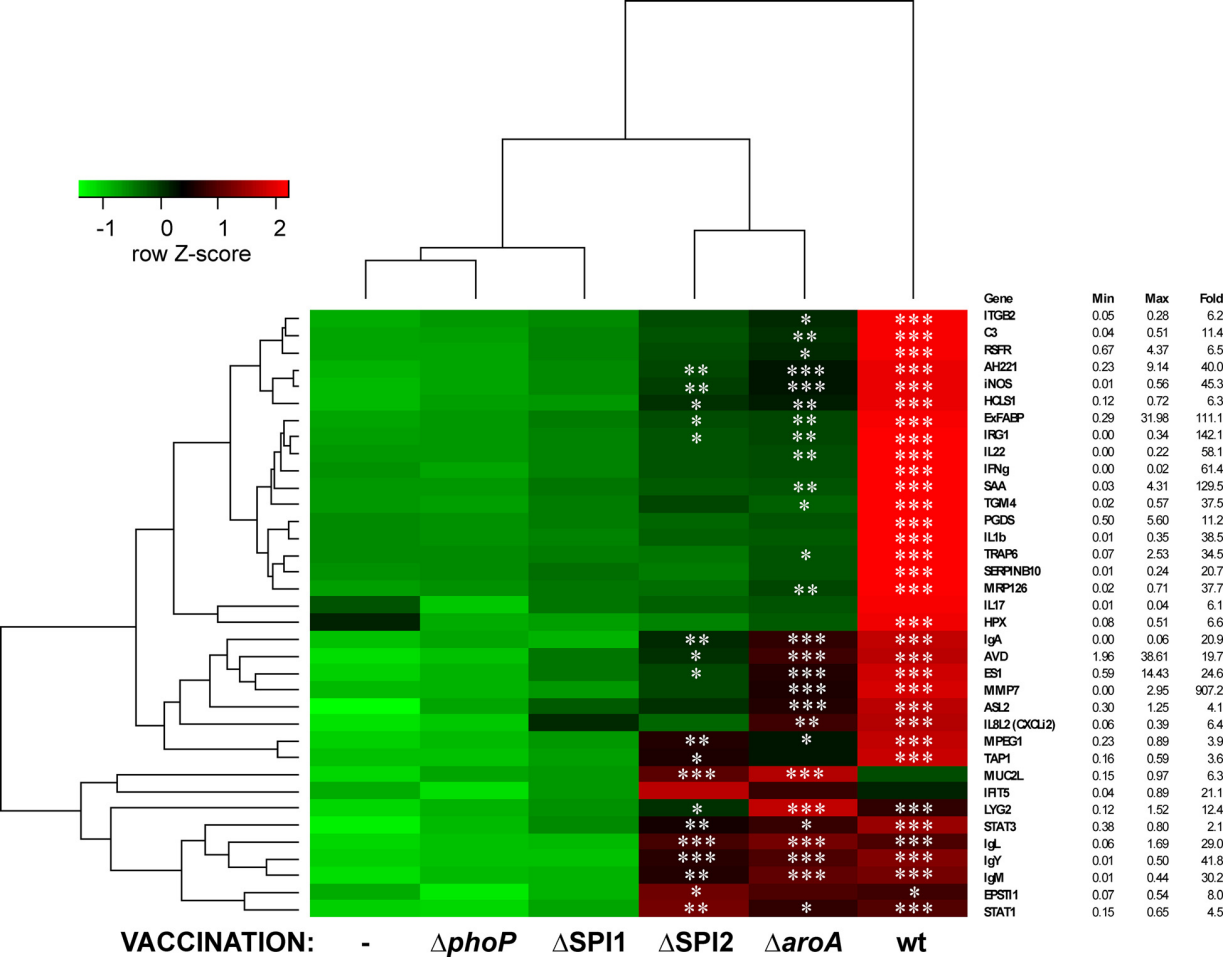
**Gene expression in chickens inoculated with wild-type** ***S.*** **Enteritidis and its mutants.** Chickens were infected on day of hatch with wild-type *S*. Enteritidis or SPI1, SPI2, *phoP* and *aroA* mutants, and euthanized 4 days later. Heat map shows average gene expression in groups of chickens. Asterisks indicate statistically significant difference from the expression in the non-infected chickens at *P* ≤ 0.05 (*), *P* ≤ 0.01 (**), or *P* ≤ 0.001 (***). Maximal fold increase is shown to highlight the differences between the green and red color range for each of the genes. Minimal and maximal expression levels are shown to remind that higher fold inductions are more frequent for genes with a lower basal expression.

Although *S*. Enteritidis counts in the cecum and liver of the chickens inoculated with different mutants did not indicate any difference in their residual virulence, differences were observed in the gene expression in the cecum. Except for MUC2L, IFIT5, LYG2, Ig λ light chain, EPSTI1 and STAT1, expression of the remaining genes was always numerically the highest in chickens infected with wild-type *S*. Enteritidis (Figure 4). Expression of MUC2L, IFIT5, LYG2, Ig λ light chain, EPSTI1 and STAT1 was the highest in chickens inoculated with either *aroA* or SPI2 mutant. In addition, SPI2 and *aroA* mutants induced 18 and 27 genes of the 36 tested in inoculated chickens, respectively. On the other hand, not a single gene out of those tested was significantly upregulated after inoculation of the chickens with the SPI1 and *phoP* mutants and the chickens inoculated with these mutants clustered with the non-inoculated control group (Figure 4). SPI1 and *phoP* mutants therefore did not stimulate an inflammatory response in inoculated chickens, SPI2 and *aroA* mutants stimulated moderate response and the highest response was induced by the wild-type *S*. Enteritidis.

### Responsiveness of chickens of different ages to *S*. Enteritidis infection

**Figure 5:**
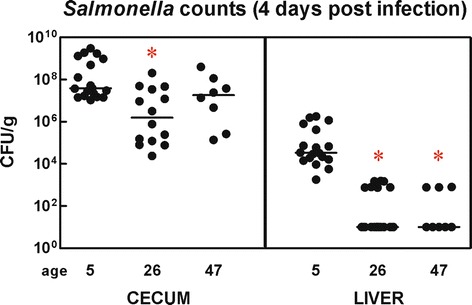
***Salmonella*** **counts in organs of chickens infected with wild-type** ***S.*** **Enteritidis at different ages.** Chickens were infected with wild-type *S*. Enteritidis on day 1, 22 or 43 of life and euthanized 4 days later. The data represent individual values and median CFU/g tissue. Asterisks indicate statistically significant difference from *Salmonella* counts in 5-day-old chickens 4 days post infection at *P* ≤ 0.05 (*).

In the next experiment we determined resistance of differently aged chickens to *S*. Enteritidis infection. *Salmonella* counts in the liver of chickens infected on day 1 were significantly higher than in chickens infected on day 22 (Figure 5). On the other hand, differences in counts of *S*. Enteritidis in the liver and cecum of chickens infected on day 22 and 43 did not reach statistical significance. When immune response was determined in the cecum, the infection of newly hatched chickens with *S*. Enteritidis led to a significantly increased expression of all tested genes with matrix metalloproteinase 7 (MMP7) being upregulated nearly 1000×. An additional 21 genes were induced more than 10× (Table 2). In 22-day-old chickens, 33 out of 36 tested genes were significantly upregulated. MMP7 was induced 139× (although not significantly due to a high variation among individual chickens) and 8 other genes (IL-22, ExFABP, IRG1, ES1, SAA, IL-17, MRP126 and AVD) were upregulated more than 10×. Infection of 43-day-old chickens led to a significant upregulation of 22 genes (Table 2). Since 22-day-old chickens were more responsive to *S*. Enteritidis infection than 43-day-old chickens, 22-day-old chickens were selected for the comparison of the immune response of naive and inoculated chickens in the following experiment.

**Table 2:** **Age-dependent responsiveness of chickens to** ***S.*** **Enteritidis infection**

**Function/gene**	**Description**	Fold increase in chickens*		
		**day 1**	**day 21**	**day 42**
**Cytokines**				
IL-1β	interleukin 1	**21.1 ± 14.4** ^**$**^	**2.6 ± 1.4**	4.2 ± 3.8
CXCLi2 (IL-8 L2)	interleukin 8	**5.9 ± 1.6**	**9.4 ± 4.9**	**7.1 ± 4.5**
IL-17	interleukin 17	**2.9 ± 1.5**	**14.4 ± 8.5**	**12.4 ± 10.3**
IL-22	interleukin 22	**50.1 ± 30.4**	**44.4 ± 27.9**	**12.3 ± 9.8**
IFNγ	interferon gamma	**20.1 ± 12.1**	**6.9 ± 3.6**	2.7 ± 1.7
AH221	chemokine AH221 (CCLi9)	**36.8 ± 7.4**	**3.6 ± 2.0**	**4.1 ± 2.5**
**Immunoglobulins**				
IgM	immunoglobulin M heavy chain, C-region	**25.8 ± 10.0**	**2.9 ± 1.0**	**1.8 ± 0.9**
IgY	imunoglobulin Y heavy chain, C-region	**44.9 ± 18.2**	**3.6 ± 1.5**	**0.9 ± 0.3**
IgA	imunoglobulin A heavy chain, C-region	**19.3 ± 11.8**	**2.9 ± 1.3**	**1.5 ± 0.4**
Ig λ	immunoglobulin lambda light chain, C-region	**25.1 ± 8.8**	**3.1 ± 1.6**	**0.6 ± 0.2**
**Other immune response proteins**				
IRG1	immune responsive gene 1	**186.2 ± 42.9**	**22.5 ± 11.5**	**25.6 ± 20.0**
iNOS	inducible NO synthase	**58.1 ± 18.1**	**5.8 ± 3.9**	**22.0 ± 19.7**
MRP126	MRP-126 (S100A9, calprotectin, calgranulin B)	**33.0 ± 13.6**	**10.1 ± 4.2**	9.3 ± 11.3
PTGDS	prostaglandin D2 synthase 21 kDa (brain)	**11.9 ± 4.0**	**2.2 ± 1.0**	**7.4 ± 5.5**
C3	complement 3	**11.4 ± 2.2**	**3.5 ± 1.1**	**2.5 ± 1.0**
IFIT5	interferon-induced protein with tetratricopeptide repeats 5	**3.6 ± 0.9**	**4.5 ± 2.5**	**8.8 ± 5.1**
ASL2	argininosuccinate lyase	**4.5 ± 1.0**	**2.6 ± 1.0**	**2.6 ± 1.2**
MPEG1	macrophage-expressed gene 1 protein-like	**4.0 ± 0.6**	**1.3 ± 0.2**	**2.6 ± 0.9**
ITGB2	integrin beta-2 precursor	**6.6 ± 1.1**	**1.6 ± 0.4**	**2.3 ± 1.0**
TAP1	transporter 1, ATP-binding cassette, sub-family B	**5.0 ± 1.4**	**2.4 ± 0.9**	**3.4 ± 1.4**
STAT1	signal transducer and activator of transcription 1	**4.1 ± 0.6**	**1.8 ± 0.4**	**3.0 ± 1.2**
STAT3	signal transducer and activator of transcription 3	**1.9 ± 0.2**	1.2 ± 0.2	**2.1 ± 0.6**
**Acute phase response**				
SAA	serum amyloid A	**150.7 ± 61.1**	**15.4 ± 9.7**	22.5 ± 22.8
AVD	avidin	**27.0 ± 8.4**	**10.1 ± 5.4**	**8.9 ± 5.9**
HPX	hemopexin	**7.0 ± 2.7**	3.0 ± 2.1	**3.5 ± 1.5**
**Mucosal defense**				
MMP7	matrix metallopeptidase 7 (matrilysin, uterine)	**939.1 ± 287.1**	138.8 ± 149.1	**12.6 ± 11.4**
ExFABP	extracellular fatty-acid binding protein (P20K, LCN8)	**177.0 ± 57.4**	**27.7 ± 14.5**	**11.6 ± 10.1**
TRAP6	trappin-6	**64.8 ± 22.5**	**3.8 ± 2.1**	10.6 ± 10.0
LYG2	lysozyme *g*-like 2	**32.9 ± 6.3**	**4.5 ± 3.1**	**15.8 ± 8.6**
MUC2L	mucin-2-like	**3.5 ± 1.3**	**8.5 ± 4.9**	2.2 ± 1.5
**Hematopoiesis, angiogenesis**				
SERPINB10	serpin peptidase inhibitor, clade B (ovalbumin), member 10	**18.0 ± 9.8**	**3.8 ± 1.5**	**8.8 ± 6.3**
HCLS1	hematopoietic lineage cell-specific protein 1	**7.8 ± 1.5**	**1.8 ± 0.6**	1.9 ± 0.8
RSFR	leucocyte ribonuclease A-2, angiogenin	**7.5 ± 1.7**	**1.7 ± 0.5**	1.9 ± 0.9
**Other**				
TGM4	glutamine γ-glutamyltransferase 4	**37.5 ± 12.0**	**2.3 ± 1.1**	9.9 ± 13.9
ES1	ES1 protein homolog	**21.4 ± 8.9**	**22.4 ± 17.8**	**17.5 ± 13.0**
EPSTI1	epithelial stromal interaction 1 (breast)	**3.6 ± 0.9**	**3.1 ± 0.9**	1.7 ± 0.8

*Chickens were infected on day 1, 21 or 42 with *S.* Enteritidis and euthanized 4 days after the infection. The table presents a fold increase in gene expression after the infection with 95% confidence interval.

^$^Values in bold indicate significant difference from the expression in age-matched non-infected control chickens.

### Response of chickens inoculated with SPI1, SPI2, *phoP* and *aroA* mutants to challenge with wild-type *S.* Enteritidis

In the last experiment we addressed whether the inoculation of newly hatched chickens with the mutants would also result in a different interaction with the wild-type *S*. Enteritidis after challenge. First we checked the colonization of 22-day-old chickens by strains used for initial inoculation. Except for 2 or 3 chickens inoculated with the SPI2 and *aroA* mutant, respectively, all the remaining chickens were free of *S*. Enteritidis in the liver. However, all the chickens, irrespective of the strain used for the inoculation on day 1 of life, were still positive for *S*. Enteritidis in the cecum (Figure 3).

Four days after the challenge, *S*. Enteritidis counts in the cecum of chickens originally inoculated with the SPI1, SPI2 and *aroA* mutant did not significantly differ from the counts in chickens which were infected with wild-type *S*. Enteritidis on day of hatch and re-infected on day 22, or which were infected only on day 22 of life (Figure 3). Only *phoP*-inoculated chickens were significantly protected against wild-type *S*. Enteritidis challenge since *S*. Enteritidis counts in the vaccinated birds were significantly lower than in the non-vaccinated controls. Differences in *Salmonella* counts in the liver were only of numerical value which did not reach statistical significance, in this case including the group inoculated with the *phoP* mutant (Figure 3).

**Figure 6:**
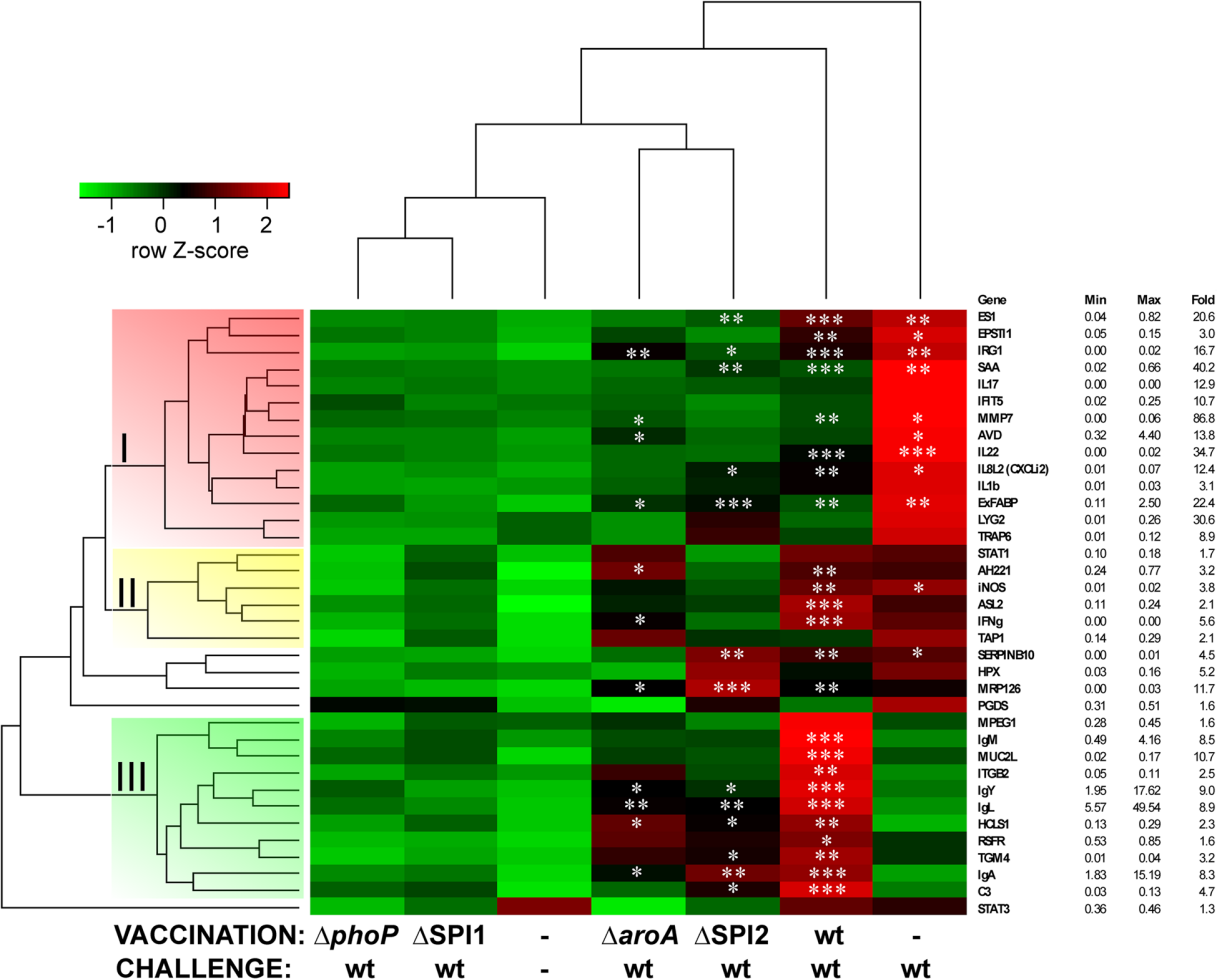
**Gene expression in inoculated chickens 4 days after challenge with wild-type** ***S*** **. Enteritidis.** Chickens were inoculated on day of hatch with SPI1, SPI2, *phoP* and *aroA S*. Enteritidis mutants, challenged on day 22 of life and euthanized 4 days later. Heat map shows average gene expression in groups of chickens. Group I of genes represents genes belonging mainly to innate immune response, group II genes belonging to Th1 cell mediated immune response and group III is mainly associated with B-lymphocyte development and antibody production. Asterisks indicate statistically significant difference from the non-infected chickens at *P* ≤ 0.05 (*), *P* ≤ 0.01 (**) or *P* ≤ 0.001 (***). Maximal fold increase is shown to highlight the differences between the green and red color range for each of the gene. Minimal and maximal expression levels are shown to remind that higher fold inductions are more frequent for genes with a lower basal expression.

However, there were clear differences when chicken gene expression profiles were compared. During the challenge experiment at 22 days of age, the responses induced by challenge with *S*. Enteritidis in birds pre-exposed to the mutants or the wild type *S*. Enteritidis were different to those birds challenged for the first time. Except for all immunoglobulin coding genes, MPEG1, TGM4, MUC2L, ITGB2, HCLS1, RSFR and C3, the naive chickens infected with the wild type *S*. Enteritidis for the first time expressed the majority of the tested genes at high levels (Figure 6).

The second group was formed by chickens inoculated on day 1 with the wild-type *S*. Enteritidis and re-infected on day 22. All immunoglobulin coding genes, MPEG1, TGM4, MUC2L, ITGB2, HCLS1, RSFR, C3, STAT1, IFNγ and ASL2 were expressed the most in chickens belonging to this group (Figure 6).

The third group was formed by chickens inoculated with the SPI2 or *aroA* mutant and challenged with the wild-type *S*. Enteritidis (Figure 6). Response of chickens vaccinated with the SPI2 or *aroA* mutant resulted in a significant upregulation of 13 or 11 genes, respectively, with IRG1, ExFABP, MRP126 (calprotectin), HCLS1, IgY, IgA and Ig λ chain being significantly induced in both groups (Figure 6).

The last group consisted of chickens inoculated with the SPI1 or *phoP* mutant and challenged with the wild-type *S*. Enteritidis. These were both protected against the challenge as not a single gene was significantly induced after the challenge with the wild-type and these chickens therefore clustered with non-infected controls (Figure 6).

## Discussion

In this study we found out that 4 tested mutants and the wild-type *S*. Enteritidis were differently recognized and processed by HD11 macrophage cell line and the chicken immune system in general. HD11 macrophages responded to the infection with the wild type *S*. Enteritidis and SPI2, *phoP* and *aroA* mutants by an increase in transcription of inflammatory genes such as IL-1β, CXCLi2 (IL-8), ExFABP, AVD, IRG1 or iNOS. Repeatedly lower induction of these genes was observed in HD11 cells infected with the SPI1 mutant. This was in contradiction with the high inflammatory signaling of porcine alveolar macrophages infected with the SPI1 mutant when compared with those infected with the wild-type *S*. Enteritidis [25]. The likely explanation is the different origin of the cell, primary porcine macrophages and cell line in the case of HD11 chicken macrophages. Behavior of HD11 macrophages was therefore dependent on SPI1-dependent invasion with the invasion deficient SPI1 mutant inducing the lowest inflammatory signaling.

Inoculation of newly hatched chickens with the SPI1 and also *phoP* mutant did not result in inflammation, which corresponds with our previous observations on vaccination with the SPI1 mutant [13]. Although the chickens at the time of challenge were still positive for the mutants used for inoculation on day 1 of life, we believe that this did not negatively affect results as it has been shown that inflammatory response decreases in chickens between the 2^nd^ and 3^rd^ week of life [22]. The fact that we did not record extensive differences in bacterial counts after challenge in different groups was likely due to an early time point for analysis, i.e. 4 days post infection. Moreover, since we did not discriminate between the counts of vaccine and challenge strains, especially the counts in the cecum have to be taken with a certain care since these could be a mixture of vaccine and challenge strains. Despite this, immune responses to challenge were quite different across all groups. Chickens inoculated with the SPI1 or *phoP* mutant were resistant to the wild-type *S*. Enteritidis challenge as this did not trigger any inflammatory response at 4 days post challenge (Figure 6). Antibody production was stimulated in the chickens inoculated with SPI2 and *aroA* mutants and challenged with the wild-type, similarly, though to a lesser extent, to chickens inoculated twice with the wild-type *S*. Enteritidis. Recently we documented that vaccination with the SPI2 mutant resulted in a higher antibody production determined by ELISA than vaccination with the SPI1 mutant [13]. However, it should be reminded that in all the experiments we used a single time point for analysis of immune response. We therefore cannot exclude a similar response to vaccination or challenge with different dynamics, i.e. we cannot exclude an earlier or delayed response of the chickens to the vaccination or challenge with different mutants or wild type *S*. Enteritidis.

The comparative approach used in this study also allowed us to address the function of individual genes involved in the chicken response to *S*. Enteritidis infection. Cluster I in Figure 6 represents genes of early response that are highly inducible in response to *S*. Enteritidis especially in non-protected chickens. These genes include ES1, IFIT5, EPSTI1, LYG2 and MMP7 expressed in cells of non-leukocyte origin [26-31], IRG1, AVD, ExFABP, SAA, IL-1β and TRAP6 expressed in macrophages and heterophils [21,32], CXCLi2 (IL-8) produced by both intestinal epithelial cells and phagocytes [33-35], and IL-17 and IL-22 expressed in T-lymphocytes [33]. Most of these genes were reported to be induced also after infections with other pathogens [36-39]. These genes can be therefore understood as being involved in the innate immune response and can be used as sensitive markers for gut inflammation in chickens.

The second cluster of genes was formed by IFNγ and AH221, iNOS, ASL2, STAT1 and TAP1 (Figure 6). These genes were expressed to a similar extent in chickens infected twice with the wild-type *S*. Enteritidis and in chickens infected with *S*. Enteritidis for the first time at the age of 22 days. However, significant induction was recorded only in re-infected chickens. Except for IFNγ produced by T-lymphocytes and NK cells, all these genes are characteristic of macrophages [21], and represent common markers of Th1 immune response characterized by NO radical production and arginine/ornithine recycling by ASL2 [40].

Group III included all 4 immunoglobulin encoding genes, MUC2L, ITGB2, HCLS1, RSFR, TGM4, MPEG1 and C3 (Figure 4). Similarly to immunoglobulins, TGM4, ITGB2 (CD18) and HCLS1 are expressed by B-lymphocytes, with ITGB2 and HCLS1 being also expressed by other hematopoietic cells [21,41-43]. All these genes were significantly induced in 22-day-old chickens only after repeated *Salmonella* infection. Some of these genes were induced also in the chickens inoculated with the *aroA* and SPI2 mutants. These genes are associated with B-lymphocyte differentiation and consequently with specific immune response and antibody production [44,45].

However, the results following the inoculation with *phoP* and SPI1 mutants were the most surprising. One would expect that if these mutants did not induce at least moderate inflammation as did the SPI2 or *aroA* mutant, specific immunity could not develop and challenged chickens should respond as the naive controls, which was not the case. The reason for the different development of specific immunity is not known. However, it is possible that due to a decreased ability to invade intestinal epithelial cells, *S*. Enteritidis SPI1 mutant should be present mainly in professional phagocytes and antigen presenting cells without being able to cause their apoptosis [24,25]. Similarly, *phoP* mutant exhibits increased intracellular replication without causing cell death [18]. It is therefore tempting to speculate that if the whole tissue is inflamed, immune response is polarized towards Th2 response and antibody production. On the other hand, if cells present in the cecal tissue are not stimulated for inflammatory signaling, the immune system is then polarized towards cell-mediated response. Although the above mentioned hypothesis will have to be proven experimentally, it can be concluded that *phoP*, *aroA*, SPI1 and SPI2 mutants were recognized and processed differently by the chicken immune system which might help in developing vaccines against either systemic or gut infection.

## Additional file

Additional file 1:
**List of primers used in real-time PCR.** Primers used for the quantification of chicken gene expression by real time PCR are listed in this file.

## References

[1] Majowicz SE, Musto J, Scallan E, Angulo FJ, Kirk M, O’Brien SJ, Jones TF, Fayil A, Hoekstra RM (2010). The global burden of nontyphoidal *Salmonella* gastroenteritis. Clin Infect Dis.

[2] Hoiseth SK, Stocker BAD (1981). Aromatic-dependent *Salmonella typhimurium* are non-virulent and effective as live vaccines. Nature.

[3] Galan JE, Curtiss R (1989). Virulence and vaccine potential of *phoP* mutants of *Salmonella typhimurium*. Microb Pathog.

[4] Khan SA, Stratford R, Wu T, McKelvie N, Bellaby T, Hindle Z, Sinha KA, Eltze S, Mastroeni P, Pickard D, Dougan G, Chatfield SN, Brennan FR (2003). *Salmonella typhi* and *S. typhimurium* derivatives harbouring deletions in aromatic biosynthesis and *Salmonella* Pathogenicity Island-2 (SPI-2) genes as vaccines and vectors. Vaccine.

[5] Bohez L, Ducatelle R, Pasmans F, Haesebrouck F, Van Immerseel F (2007). Long-term colonisation-inhibition studies to protect broilers against colonisation with *Salmonella* Enteritidis, using *Salmonella* Pathogenicity Island 1 and 2 mutants. Vaccine.

[6] Rychlik I, Karasova D, Sebkova A, Volf J, Sisak F, Havlickova H, Kummer V, Imre A, Szmolka A, Nagy B (2009). Virulence potential of five major pathogenicity islands (SPI-1 to SPI-5) of *Salmonella enterica* serovar Enteritidis for chickens. BMC Microbiol.

[7] Hohmann EL, Oletta CA, Killeen KP, Miller SI (1996). phoP/phoQ-deleted *Salmonella typhi* (Ty800) is a safe and immunogenic single-dose typhoid fever vaccine in volunteers. J Infect Dis.

[8] Raupach B, Kaufmann SHE (2001). Bacterial virulence, proinflammatory cytokines and host immunity: how to choose the appropriate *Salmonella* vaccine strain?. Microb Infect.

[9] Sebkova A, Karasova D, Crhanova M, Budinska E, Rychlik I (2008). *aroA* mutations in *Salmonella enterica* cause defects in cell wall and outer membrane integrity. J Bacteriol.

[10] Fields PI, Groisman EA, Heffron F (1989). *Salmonella* locus that controls resistance to microbicidal proteins from phagocytic cells. Science.

[11] Bader MW, Navarre WW, Shiau W, Nikaido H, Frye JG, McClelland M, Fang FC, Miller SI (2003). Regulation of *Salmonella typhimurium* virulence gene expression by cationic antimicrobial peptides. Mol Microbiol.

[12] Nunez-Hernandez C, Tierrez A, Ortega AD, Pucciarelli MG, Godoy M, Eisman B, Casadesus J, Garcia-del Portillo F (2013). Genome expression analysis of nonproliferating intracellular *Salmonella enterica* serovar Typhimurium unravels an acid pH-dependent PhoP-PhoQ response essential for dormancy. Infect Immun.

[13] Matulova M, Havlickova H, Sisak S, Rychlik I (2012). Vaccination of chickens with *Salmonella* Pathogenicity Island (SPI) 1 and SPI2 defective mutants of *Salmonella enterica* serovar Enteritidis. Vaccine.

[14] Galan JE (1999). Interaction of *Salmonella* with host cells through the centisome 63 type III secretion system. Curr Opin Microbiol.

[15] Ochman H, Soncini FC, Solomon F, Groisman EA (1996). Identification of a pathogenicity island required for *Salmonella* survival in host cells. Proc Natl Acad Sci U S A.

[16] Cirillo DM, Valdivia RH, Monack DM, Falkow S (1998). Macrophage-dependent induction of the *Salmonella* pathogenicity island 2 type III secretion system and its role in intracellular survival. Mol Microbiol.

[17] Hensel M, Shea JE, Waterman SR, Mundy R, Nikolaus T, Banks G, Vazquez-Torres A, Gleeson C, Fang FC, Holden DW (1998). Genes encoding putative effector proteins of the type III secretion system of *Salmonella* pathogenicity island 2 are required for bacterial virulence and proliferation in macrophages. Mol Microbiol.

[18] Lundberg U, Vinatzer U, Berdnik D, von Gabain A, Baccarini M (1999). Growth phase-regulated induction of *Salmonella*-induced macrophage apoptosis correlates with transient expression of SPI-1 genes. J Bacteriol.

[19] Hersh D, Monac DM, Smith MR, Ghori N, Falkow S, Zychlinsky A (1999). The *Salmonella* invasin SipB induces macrophages apoptosis by binding to caspase-1. Proc Natl Acad Sci U S A.

[20] Beal RK, Wigley P, Powers C, Hulme SD, Barrow PA, Smith AL (2004). Age at primary infection with *Salmonella enterica* serovar Typhimurium in the chicken influences persistence of infection and subsequent immunity to re-challenge. Vet Immunol Immunopathol.

[21] Matulova M, Rajova J, Vlasatikova L, Volf J, Stepanova H, Havlickova H, Sisak F, Rychlik I (2012). Characterization of chicken spleen transcriptome after infection with *Salmonella enterica* serovar Enteritidis. PLoS One.

[22] Matulova M, Varmuzova K, Sisak F, Havlickova H, Babak V, Stejskal K, Zdrahal Z, Rychlik I (2013). Chicken innate immune response to oral infection with *Salmonella enterica* serovar Enteritidis. Vet Res.

[23] Matulova M, Havlickova H, Sisak F, Babak V, Rychlik I (2013). SPI1 defective mutants of *Salmonella enterica* induce cross-protective immunity in chickens against challenge with serovars Typhimurium and Enteritidis. Vaccine.

[24] Karasova D, Sebkova A, Havlickova H, Sisak F, Volf J, Faldyna M, Ondrackova PV, Kummer V, Rychlik I (2010). Influence of 5 major *Salmonella* pathogenicity islands on NK cell depletion in mice infected with *Salmonella enterica* serovar Enteritidis. BMC Microbiol.

[25] Pavlova B, Volf J, Ondrackova P, Matiasovic J, Stepanova H, Crhanova M, Karasova D, Faldyna M, Rychlik I (2011). SPI-1-encoded type III secretion system of *Salmonella enterica* is required for the suppression of porcine alveolar macrophage cytokine expression. Vet Res.

[26] Shin JH, Weitzdoerfer R, Fountoulakis M, Lubec G (2004). Expression of cystathionine β-synthase, pyridoxal kinase, and ES1 protein homolog (mitochondrial precursor) in fetal Down syndrome brain. Neurochem Int.

[27] Katibah GE, Lee HJ, Huizar JP, Vogan JM, Alber T, Collins K (2013). tRNA Binding, structure, and localization of the human interferon-induced protein IFIT5. Mol Cell.

[28] Ovstebo R, Olstad OK, Brusletto B, Moller AS, Aase A, Haug KB, Brandtzaeg P, Kierulf P (2008). Identification of genes particularly sensitive to lipopolysaccharide (LPS) in human monocytes induced by wild-type versus LPS-deficient *Neisseria meningitidis* strains. Infect Immun.

[29] de Neergaard M, Kim J, Villadsen R, Fridriksdottir AJ, Rank F, Timmermans-Wielenga V, Langerod A, Borrensen-Dale AL, Petersen OW, Rønnov-Jessen L (2010). Epithelial-Stromal Interaction 1 (EPSTI1) Substitutes for peritumoral fibroblasts in the tumor microenvironment. Am J Pathol.

[30] Scherer RL, VanSaun MN, McIntyre JO, Matrisian LM (2008). Optical imaging of matrix metalloproteinase-7 activity in vivo using a proteolytic nanobeacon. Mol Imaging.

[31] Nile CJ, Townes CL, Michailidis G, Hirst BH, Hall J (2004). Identification of chicken lysozyme g2 and its expression in the intestine. Cell Mol Life Sci.

[32] Desin B, Descalzi F, Briata L, Hayashi M, Gentili C, Hayashi K, Quarto R, Cancedda R (1992). Expression, regulation, and tissue distribution of the Ch21 protein during chicken embryogenesis. J Biol Chem.

[33] Matulova M, Stepanova H, Sisak F, Havlickova H, Faldynova M, Kyrova K, Volf J, Rychlik I (2012). Cytokine signaling in splenic leukocytes from vaccinated and non-vaccinated chickens after intravenous infection with *Salmonella* Enteritidis. PLoS One.

[34] Salisbury AM, Bronowski C, Wigley P (2011). *Salmonella* Virchow isolates from human and avian origins in England--molecular characterization and infection of epithelial cells and poultry. J Appl Microbiol.

[35] Kogut MH, Genovese KJ, He H, Kaiser P (2008). Flagellin and lipopolysaccharide up-regulation of IL-6 and CXCLi2 gene expression in chicken heterophils is mediated by ERK1/2-dependent activation of AP-1 and NF-kappa B signaling pathways. Innate Immun.

[36] Zhang B, Liu X, Chen W, Chen L (2013). IFIT5 potentiates anti-viral response through enhancing innate immune signaling pathways. Acta Biochim Biophys Sin (Shanghai).

[37] Lopez-Boado YS, Wilson CL, Hooper LV, Gordon JI, Hultgren SJ, Parks WC (2000). Bacterial exposure induces and activates matrilysin in mucosal epithelial cells. J Cell Biol.

[38] Basler T, Jeckstadt S, Valentin-Weigand P, Goethe R (2006). *Mycobacterium paratuberculosis*, *Mycobacterium smegmatis*, and lipopolysaccharide induce different transcriptional and post-transcriptional regulation of the IRG1 gene in murine macrophages. J Leukoc Biol.

[39] Kunnas TA, Wallen MJ, Kulomaa MS (1993). Induction of chicken avidin and related messenger-RNAs after bacterial-infection. Biochem Biophys Acta.

[40] Wu G, Morris SM (1998). Arginine metabolism: nitric oxide and beyond. Biochem J.

[41] Tohma S, Hirohata S, Lipsky PE (1991). The role of CD11a/CD18-CD54 interactions in human T cell-dependent B cell activation. J Immunol.

[42] Kitamura D, Kaneko H, Miyagoe Y, Ariyasu T, Watanabe T (1989). Isolation and characterization of a novel human gene expressed specifically in the cells of hematopoietic lineage. Nucl Acids Res.

[43] Yamanashi Y, Okada M, Semba T, Yamori T, Umemori H, Tsunasawa S, Toyoshima K, Kitamura D, Watanabe T, Yamamoto T (1993). Identification of HS1 protein as a major substrate of protein-tyrosine kinase(s) upon B-cell antigen receptor-mediated signaling. Proc Natl Acad Sci U S A.

[44] Hao J, Carey GB, Zhan X (2004). Syk-mediated tyrosine phosphorylation is required for the association of hematopoietic lineage cell-specific protein 1 with lipid rafts and B cell antigen receptor signalosome complex. J Biol Chem.

[45] Taniuchi I, Kitamura D, Maekawa Y, Fukuda T, Kishi H, Watanabe T (1995). Antigen-receptor induced clonal expansion and deletion of lymphocytes are impaired in mice lacking HS1 protein, a substrate of the antigen-receptor-coupled tyrosine kinases. EMBO J.

[46] Karasova D, Sebkova A, Vrbas V, Havlickova H, Sisak F, Rychlik I (2009). Comparative analysis of *Salmonella enterica* serovar Enteritidis mutants with a vaccine potential. Vaccine.

